# Supplementary material for: Current status of intestinal parasitosis and microsporidiosis in industrialized countries: Results from a prospective study in France and Luxembourg
Source: PLoS Negl Trop Dis. 2024 Dec 23;18(12):e0012752. doi: 10.1371/journal.pntd.0012752 (PMC11706478; doi:10.1371/journal.pntd.0012752)
Supplement: S2 Table — (DOCX) [file pntd.0012752.s002.docx]

**S2 Table. Prevalence by parasite according to the diagnostic method used.**

|  | **Microscopy** | |  | **Molecular biology** | |
| --- | --- | --- | --- | --- | --- |
|  | **Number of positive patients** | **Prevalence**  **% (95%CI)** |  | **Number of positive patients** | **Prevalence**  **% (95%CI)** |
| *Blastocystis* sp*.* | 110 | 7.0 (5.8; 8.4) |  | 293 | 18.7 (16.8; 20.7) |
| *Dientamoeba fragilis* | − | − |  | 206 | 13.1 (11.5; 14.9) |
| *Giardia intestinalis* | 13 | 0.8 (0.4; 1.4) |  | 30 | 1.9 (1.3; 2.7) |
| *Cryptosporidium* sp*.* | 6 | 0.4 (0.1; 0.8) |  | 30 | 1.9 (1.3; 2.7) |
| *Enterobius vermicularis* | 5 | 0.3 (0.1; 0.7) |  | 28 | 1.8 (1.2; 2.6) |
| *Entamoeba coli* | 23 | 1.5 (0.9; 2.2) |  | − | − |
| *Endolimax nana* | 19 | 1.2 (0.7; 1.9) |  | − | − |
| *Entamoeba dispar* | 5 | 0.3 (0.1; 0.7) |  | 17 | 1.1 (0.6; 1.7) |
| Microsporidia* | − | − |  | 7 | 0.4 (0.2; 0.9) |
| *Taenia* sp*.* | 3 | 0.2 (0.0; 0.6) |  | 5 | 0.3 (0.1; 0.7) |
| *Strongyloides stercoralis* | 0 | 0 |  | 4 | 0.3 (0.1; 0.7) |
| *Cystoisospora belli* | 0 | 0 |  | 3 | 0.2 (0.0; 0.6) |
| *Dicrocoelium* sp*.* | 2 | 0.1 (0.0; 0.5) |  | − | − |
| *Entamoeba hartmanni* | 2 | 0.1 (0.0; 0.5) |  | − | − |
| *Iodamoeba butschlii* | 2 | 0.1 (0.0; 0.5) |  | − | − |
| *Schistosoma mansoni* | 0 | 0 |  | 2 | 0.1 (0.0; 0.5) |
| *Chilomastix mesnili* | 1 | 0.1 (0.0; 0.4) |  | − | − |
| *Sarcocystis* sp*.* | 1 | 0.1 (0.0; 0.4) |  | − | − |
| *Cyclospora cayetanensis* | 0 | 0 |  | 1 | 0.1 (0.0; 0.4) |
| *Hymenolepis nana* | 1 | 0.1 (0.0; 0.4) |  | 1 | 0.1 (0.0; 0.4) |
| *Trichuris trichiura* | 1 | 0.1 (0.0; 0.4) |  | 1 | 0.1 (0.0; 0.4) |

Results are presented by patient (n=1570). Parasites are sorted from most to least prevalent. Dashes are used when a parasite cannot be detected by one diagnostic method or another. Data not shown are equal to zero. CI: confidence interval. **Enterocytozoon* *bieneusi* + *Encephalitozoon* sp
