# Supplementary material for: Current status of intestinal parasitosis and microsporidiosis in industrialized countries: Results from a prospective study in France and Luxembourg
Source: PLoS Negl Trop Dis. 2024 Dec 23;18(12):e0012752. doi: 10.1371/journal.pntd.0012752 (PMC11706478; doi:10.1371/journal.pntd.0012752)
Supplement: S3 Table — (DOCX) [file pntd.0012752.s003.docx]

**S3 Table.** **Parasite and microsporidiosis prevalence by microscopy and molecular biology according to the number of stool samples per patient.**

|  | **Microscopy + molecular biology** | | | | | | **Microscopy** | | | |  | **Molecular biology** | | | | | | | | |
| --- | --- | --- | --- | --- | --- | --- | --- | --- | --- | --- | --- | --- | --- | --- | --- | --- | --- | --- | --- | --- |
|  | **1**  **(n=1270)** | **2**  **(n=145)** | **≥ 3**  **(n=155)** | **p** |  | **1**  **(n=1270)** | | **2**  **(n=145)** | **≥ 3**  **(n=155)** | **p** | | |  | **1**  **(n=1270)** | **2**  **(n=145)** | **≥ 3**  **(n=155)** | **p** |  |  |  |
| Overall positivity | 395 (31.1) | 58 (40.0) | 68 (43.9) | **0.001** |  | 114 (9.0) | | 27 (18.62) | 27 (17.42) | **<0.001** | | |  | 372 (29.3) | 54 (37.2) | 64 (41.29) | **0.003** |  |  |  |
| *Blastocystis* sp*.* | 239 (18.8) | 38 (26.2) | 45 (29.0) | **0.002** |  | 72 (5.7) | | 17 (11.7) | 21 (13.6) | **<0.001** | | |  | 221 (17.4) | 34 (23.5) | 38 (24.5) | **0.03** |  |  |  |
| *Dientamoeba fragilis* | 157 (12.4) | 24 (16.6) | 25 (16.1) | 0.19 |  | - | | - | - | - | | |  | 157 (12.4) | 24 (16.6) | 25 (16.1) | 0.19 |  |  |  |
| *Giardia intestinalis* | 25 (2.0) | 2 (1.4) | 3 (2.0) | 1.00 |  | 11 (0.9) | | 1 (0.7) | 1 (0.7) | 1.00 | | |  | 25 (2.0) | 2 (1.4) | 3 (2.0) | 1.00 |  |  |  |
| *Cryptosporidium* sp*.* | 22 (1.7) | 4 (2.8) | 4 (2.6) | 0.44 |  | 5 (0.4) | | 1 (0.7) | 0 (0.0) | 0.51 | | |  | 22 (1.7) | 4 (2.8) | 4 (2.6) | 0.44 |  |  |  |
| *Enterobius vermicularis* | 21 (1.7) | 3 (2.1) | 5 (3.2) | 0.32 |  | 2 (0.2) | | 2 (1.4) | 1 (0.7) | **0.03** | | |  | 20 (1.6) | 3 (2.1) | 5 (3.2) | 0.29 |  |  |  |
| *Entamoeba coli* | 17 (1.3) | 5 (3.5) | 1 (0.7) | 0.10 |  | 17 (1.3) | | 5 (3.5) | 1 (0.7) | 0.10 | | |  | - | - | - | - |  |  |  |
| *Endolimax nana* | 13 (1.0) | 1 (0.7) | 5 (3.2) | 0.06 |  | 13 (1.0) | | 1 (0.7) | 5 (3.2) | 0.06 | | |  | - | - | - | - |  |  |  |
| *Entamoeba dispar* | 11 (0.9) | 5 (3.5) | 1 (0.7) | **0.03** |  | 2 (0.2) | | 2 (1.4) | 1 (0.7) | **0.03** | | |  | 11 (0.9) | 5 (3.5) | 1 (0.7) | **0.03** |  |  |  |
| Microsporidia* | 5 (0.4) | 1 (0.7) | 1 (0.7) | 0.72 |  | - | | - | - | - | | |  | 5 (0.4) | 1 (0.7) | 1 (0.7) | 0.72 |  |  |  |
| *Taenia* sp. | 3 (0.2) | 2 (1.4) | 0 (0.0) | 0.10 |  | 2 (0.2) | | 1 (0.7) | 0 (0.0) | 0.28 | | |  | 3 (0.2) | 2 (1.4) | 0 (0.0) | 0.10 |  |  |  |

Data are presented as number of patients (percentage). Total number of patients is 1570. To help reading, p-values <0.05 are indicated in bold. **Enterocytozoon* *bieneusi* + *Encephalitozoon* sp.
