# Supplementary material for: Current status of intestinal parasitosis and microsporidiosis in industrialized countries: Results from a prospective study in France and Luxembourg
Source: PLoS Negl Trop Dis. 2024 Dec 23;18(12):e0012752. doi: 10.1371/journal.pntd.0012752 (PMC11706478; doi:10.1371/journal.pntd.0012752)
Supplement: S4 Table — (DOCX) [file pntd.0012752.s004.docx]

**S4 Table.** **Number of stool samples required to detect all coinfections** **in the subset of patients with three stool samples according to the diagnostic method used.**

| **Co-occurrence** | | **Case number** | **Microscopy +  molecular biology** | **Microscopy** | **Molecular biology** |
| --- | --- | --- | --- | --- | --- |
| *Blastocystis* sp. +  *Dientamoeba fragilis* | (n=8) | 294 | 1^st^ | n.a. | n.d. |
|  |  | 378 | 1^st^ | n.a. | 1^st^ |
|  |  | 445 | 1^st^ | n.a. | 1^st^ |
|  |  | 457 | 1^st^ | n.a. | 1^st^ |
|  |  | 505 | 3^rd^ | n.a. | 3^rd^ |
|  |  | 507 | 3^rd^ | n.a. | 3^rd^ |
|  |  | 614 | 1^st^ | n.a. | n.d. |
|  |  | 1314 | 3^rd^ | n.a. | n.d. |
| *Dientamoeba fragilis* +  *Enterobius vermicularis* | (n=4) | 90 | 3^rd^ | n.a. | 3^rd^ |
|  |  | 221 | 1^st^ | n.a. | 1^st^ |
|  |  | 248 | 2^nd^ | n.a. | 2^nd^ |
|  |  | 506 | 3^rd^ | n.a. | 3^rd^ |
| *Blastocystis* sp. +  *Endolimax nana* | (n=4) | 763 | 1^st^ | 1^st^ | n.a. |
|  |  | 838 | 1^st^ | n.d. | n.a. |
|  |  | 1451 | 1^st^ | 1^st^ | n.a. |
|  |  | 1592 | 2^nd^ | n.d. | n.a. |
| *Blastocystis* sp. +  *Giardia intestinalis* | (n=2) | 730 | 2^nd^ | n.d. | 2^nd^ |
|  |  | 1625 | 1^st^ | n.d. | 1^st^ |
| *Endolimax nana + Enterobius vermicularis* | (n=1) | 1325 | 2^nd^ | n.d. | n.a. |
| *Entamoeba coli + Entamoeba dispar* | (n=1) | 1423 | 2^nd^ | 3^rd^ | n.a. |
| *Blastocystis* sp. +  *Dientamoeba fragilis+ Cryptosporidium* sp. | (n=1) | 1669 | 2^nd^ | n.a. | 2^nd^ |

This table indicates whether all parasites of a co-occurrence were diagnosed in the first, second, or third stool sample. Dashes are used when a parasite, and thus co-occurrence, cannot be detected by one diagnostic method or another. n.a.: not applicable (at least one parasite of the co-occurrence cannot be detected by this method); n.d.: not detected (at least one parasite of the co-occurrence is missing).
