# Supplementary material for: Current status of intestinal parasitosis and microsporidiosis in industrialized countries: Results from a prospective study in France and Luxembourg
Source: PLoS Negl Trop Dis. 2024 Dec 23;18(12):e0012752. doi: 10.1371/journal.pntd.0012752 (PMC11706478; doi:10.1371/journal.pntd.0012752)
Supplement: S5 Table — (DOCX) [file pntd.0012752.s005.docx]

**S5 Table. Multivariable analysis of *Blastocystis* sp. and *Dientamoeba fragilis* carriage.**

|  | ***Blastocystis* sp*.*** | |  | ***Dientamoeba fragilis*** | |
| --- | --- | --- | --- | --- | --- |
|  | **OR (95%CI)** | **p** |  | **OR (95%CI)** | **p** |
| Sex |  |  |  |  |  |
| Female | Ref. |  |  | Ref. |  |
| Male | 1.28 (0.99; 1.64) | 0.06 |  | 0.95 (0.69; 1.31) | 0.75 |
| Region |  |  |  |  |  |
| North | Ref. |  |  | Ref. |  |
| South | 0.83 (0.64; 1.08) | 0.17 |  | 1.25 (0.90; 1.72) | 0.19 |
| Season |  |  |  |  |  |
| Winter | Ref. |  |  | Ref. |  |
| Summer | 1.05 (0.82; 1.35) | 0.70 |  | 0.69 (0.50; 0.96) | 0.03 |
| Age (years) |  |  |  |  |  |
| <5 | Ref. |  |  | Ref. |  |
| 5-14 | 3.25 (1.49; 7.10) | 0.003 |  | 4.17 (2.32; 7.49) | <0.001 |
| 15-24 | 2.27 (1.05; 4.90) | 0.04 |  | 0.52 (0.26; 1.02) | 0.06 |
| 25-44 | 3.09 (1.55; 6.17) | 0.001 |  | 0.53 (0.30; 0.92) | 0.03 |
| ≥45 | 3.38 (1.73; 6.62) | <0.001 |  | 0.38 (0.23; 0.65) | <0.001 |

The other parasites were not present in sufficient numbers to perform multivariable analysis. OR: odds ratio, CI: confidence interval, Ref.: reference.
