# Supplementary material for: Current status of intestinal parasitosis and microsporidiosis in industrialized countries: Results from a prospective study in France and Luxembourg
Source: PLoS Negl Trop Dis. 2024 Dec 23;18(12):e0012752. doi: 10.1371/journal.pntd.0012752 (PMC11706478; doi:10.1371/journal.pntd.0012752)
Supplement: S6 Table — (DOCX) [file pntd.0012752.s006.docx]

**S6 Table.** **Parasites and microsporidia prevalence by microscopy and molecular biology according to sex, geographical origin, season of sampling and age.**

|  | **Sex** | | |  | **Region** | | |  | **Season** | | |  | **Age (years)** | | | | | |
| --- | --- | --- | --- | --- | --- | --- | --- | --- | --- | --- | --- | --- | --- | --- | --- | --- | --- | --- |
|  | **Female**  **(n=899)** | **Male**  **(n=671)** | **p** |  | **North**  **(n=983)** | **South**  **(n=582)** | **p** |  | **Winter**  **(n=840)** | **Summer**  **(n=730)** | **p** |  | **<5**  **(n=120)** | **5-14**  **(n=115)** | **15-24**  **(n=169)** | **25-44**  **(n=413)** | **≥45**  **(n=752)** | **p** |
| **Microscopy** |  |  |  |  |  |  |  |  |  |  |  |  |  |  |  |  |  |  |
| Overall positivity | 89 (9.9) | 79 (11.8) | 0.24 |  | 102 (10.4) | 66 (11.3) | 0.55 |  | 90 (10.7) | 78 (10.7) | 0.99 |  | 5 (4.2) | 16 (13.9) | 16 (9.5) | 44 (10.7) | 87 (11.6) | 0.11 |
| *Blastocystis* sp*.* | 62 (6.9) | 48 (7.2) | 0.84 |  | 67 (6.8) | 43 (7.4) | 0.67 |  | 58 (6.9) | 52 (7.1) | 0.87 |  | 2 (1.7) | 9 (7.8) | 10 (5.9) | 24 (5.8) | 6 (8.6) | **0.046** |
| *Dientamoeba fragilis* | - | - | - |  | - | - | - |  | - | - | - |  | - | - | - | - | - | - |
| *Giardia intestinalis* | 4 (0.4) | 9 (1.3) | 0.053 |  | 5 (0.5) | 8 (1.4) | 0.09 |  | 7 (0.8) | 6 (0.8) | 0.98 |  | 0 (0.0) | 4 (3.5) | 1 (0.6) | 3 (0.7) | 5 (0.7) | 0.08 |
| *Cryptosporidium* sp*.* | 2 (0.2) | 4 (0.6) | 0.41 |  | 5 (0.5) | 1 (0.2) | 0.42 |  | 1 (0.1) | 5 (0.7) | 0.10 |  | 2 (1.7) | 1 (0.9) | 2 (1.2) | 1 (0.2) | 0 (0.0) | **0.005** |
| *Enterobius vermicularis* | 3 (0.3) | 2 (0.3) | n.d. |  | 2 (0.2) | 3 (0.5) | n.d. |  | 3 (0.4) | 2 (0.3) | n.d. |  | 0 (0.0) | 2 (1.7) | 1 (0.6) | 0 (0.0) | 2 (0.3) | n.d. |
| *Entamoeba coli* | 8 (0.9) | 15 (2.2) | **0.03** |  | 18 (1.8) | 5 (0.9) | 0.12 |  | 17 (2.0) | 6 (0.8) | **0.048** |  | 0 (0.0) | 3 (2.6) | 1 (0.6) | 8 (1.9) | 11 (1.5) | 0.37 |
| *Endolimax nana* | 9 (1.0) | 10 (1.5) | 0.38 |  | 9 (0.9) | 10 (1.7) | 0.16 |  | 7 (0.8) | 12 (1.6) | 0.14 |  | 0 (0.0) | 1 (0.9) | 2 (1.2) | 9 (2.2) | 7 (0.9) | 0.30 |
| *Entamoeba dispar* | 2 (0.3) | 3 (1.3) | n.d. |  | 4 (0.4) | 1 (0.2) | n.d. |  | 2 (0.2) | 3 (0.4) | n.d. |  | 0 (0.0) | 0 (0.0) | 1 (0.6) | 3 (0.7) | 1 (0.1) | n.d. |
| Microsporidia* | - | - | - |  | - | - | - |  | - | - | - |  | - | - | - | - | - | - |
| **Molecular biology** |  |  |  |  |  |  |  |  |  |  |  |  |  |  |  |  |  |  |
| Overall positivity | 269 (29.9) | 221 (32.9) | 0.20 |  | 308 (31.3) | 179 (30.8) | 0.81 |  | 263 (31.3) | 227 (31.1) | 0.93 |  | 36 (30.0) | 68 (59.1) | 50 (29.6) | 127 (30.8) | 209 (27.8) | **<0.001** |
| *Blastocystis* sp*.* | 156 (17.4) | 137 (20.4) | 0.12 |  | 193 (19.6) | 99 (17.0) | 0.20 |  | 153 (18.2) | 140 (19.2) | 0.63 |  | 10 (8.3) | 26 (22.6) | 26 (15.4) | 79 (19.1) | 152 (20.2) | **0.02** |
| *Dientamoeba fragilis* | 111 (12.3) | 95 (14.2) | 0.29 |  | 122 (12.4) | 83 (14.3) | 0.29 |  | 126 (15.0) | 80 (11.0) | **0.02** |  | 23 (19.2) | 57 (49.6) | 18 (10.7) | 45 (10.9) | 63 (8.4) | **<0.001** |
| *Giardia intestinalis* | 15 (1.7) | 15 (2.2) | 0.46 |  | 13 (1.3) | 16 (2.8) | **0.04** |  | 11 (1.3) | 19 (2.6) | 0.06 |  | 1 (0.8) | 5 (4.4) | 3 (1.8) | 6 (1.5) | 15 (2.0) | 0.36 |
| *Cryptosporidium* sp*.* | 14 (1.6) | 16 (2.4) | 0.24 |  | 18 (1.8) | 12 (2.1) | 0.75 |  | 8 (1.0) | 22 (3.0) | **0.003** |  | 3 (2.5) | 4 (3.5) | 4 (2.4) | 5 (1.2) | 14 (1.9) | 0.44 |
| *Enterobius vermicularis* | 15 (1.7) | 13 (1.9) | 0.69 |  | 18 (1.8) | 10 (1.7) | 0.87 |  | 19 (2.3) | 9 (1.2) | 0.12 |  | 4 (3.3) | 7 (6.1) | 6 (3.6) | 5 (1.2) | 6 (0.8) | **<0.001** |
| *Entamoeba coli* | - | - | - |  | - | - | - |  | - | - | - |  | - | - | - | - | - | - |
| *Endolimax nana* | - | - | - |  | - | - | - |  | - | - | - |  | - | - | - | - | - | - |
| *Entamoeba dispar* | 4 (0.4) | 13 (1.9) | **0.005** |  | 10 (1.0) | 7 (1.2) | 0.73 |  | 9 (1.1) | 8 (1.1) | 0.96 |  | 0 (0.0) | 0 (0.0) | 3 (1.8) | 8 (1.9) | 6 (0.8) | 0.18 |
| Microsporidia* | 4 (0.4) | 3 (0.4) | 1.00 |  | 6 (0.6) | 0 (0.0) | 0.09 |  | 3 (0.4) | 4 (0.5) | 0.71 |  | 1 (0.8) | 0 (0.0) | 1 (0.6) | 1 (0.2) | 4 (0.5) | 0.72 |

Data are presented as number of patients (percentage). Total number of patients is 1570 for sex and season, 1569 for age group (one birthdate lacking), and 1565 for geographical origin (five ZIP code lacking, see Fig 2 for the definition of North and South areas). p-values were calculated only for the most commonly represented parasites and microsporidia. Dashes are used when a parasite or microsporidia cannot be detected by one diagnostic method or another. To help reading, p-values <0.05 are indicated in bold. n.d.: not determined. **Enterocytozoon* *bieneusi* + *Encephalitozoon* sp.
