# Supplementary material for: Current status of intestinal parasitosis and microsporidiosis in industrialized countries: Results from a prospective study in France and Luxembourg
Source: PLoS Negl Trop Dis. 2024 Dec 23;18(12):e0012752. doi: 10.1371/journal.pntd.0012752 (PMC11706478; doi:10.1371/journal.pntd.0012752)
Supplement: Attachment — Submitted filename: Cover letter_Parasitosis in France_PNTD_revised_PP_CN.docx [file pntd.0012752.s008.docx]

<https://www.google.fr/url?sa=i&url=https://twitter.com/chuclermontfd?lang%3Deu&psig=AOvVaw2bG-aOKDB22am21JcCrsHh&ust=1610965014361000&source=images&cd=vfe&ved=0CAIQjRxqFwoTCIjTu73eou4CFQAAAAAdAAAAABAE>16 October 2024

**Cover letter**

**SERVICE DE**

**PARASITOLOGIE-MYCOLOGIE**

***C****entre* ***N****ational de* ***R****éférence*

*des* ***C****ryptosporidioses,* ***M****icrosporidies*

*et* ***A****utres* ***P****rotozooses digestives (****CMAP****)*

**Pr Philippe POIRIER - Chef de Service**

Professeur des Universités – Praticien Hospitalier

[ppoirier@chu-clermontferrand.fr](mailto:ppoirier@chu-clermontferrand.fr)

**Mme Magali BOURG - Cadre de Santé**

mbourg@chu-clermontferrand.fr

**Mme Carine BONALDI - Secrétaire**

[cbonaldi@chu-clermontferrand.fr](mailto:cbonaldi@chu-clermontferrand.fr)

**Dr Céline NOURRISSON**

Maître de Conférences des Universités – Praticien Hospitalier

[c_nourrisson@chu-clermontferrand.fr](mailto:c_nourrisson@chu-clermontferrand.fr)

**Pr Valérie LIVRELLI**

Professeur des Universités

Praticien Attaché

[vlivrelli@chu-clermontferrand.fr](mailto:vlivrelli@chu-clermontferrand.fr)

**Dr Maxime MONIOT**

Praticien Hospitalier

[mmoniot@chu-clermontferrand.fr](mailto:mmoniot@chu-clermontferrand.fr)

**Mme Patricia COMBES**

Ingénieure CNR CMAP

pcombes@chu-clermontferrand.fr

Service de Parasitologie-Mycologie
Centre de biologie (5ème étage)
CHU Gabriel Montpied
58 rue Montalembert
63003 Clermont-Ferrand
Cedex 1

Tel: 33 (0)4 73 75 01 95
Fax: 33 (0)4 73 75 01 96

Dear Editorial team,

First of all, we would like to thank the editor for considering our article PNTD-D-24-01049 and the reviewers for their constructive comments. Please find attached the revised manuscript which we should like to submit for publication in *PLOS Neglected Tropical Diseases*.

As expected, we have addressed below each of the issues raised by the reviewers in a point-by-point reply (in bold). The modifications in the new version of the manuscript are highlighted in yellow.

We hope that changes that have been made in our amended manuscript will answer to all reviewers’ queries and that you will find our article suitable for publication in your journal. All authors agree with this new version of our manuscript.

Yours faithfully,

Pr Philippe Poirier

Reviewer #1:

Line 112, preserved or unpreserved samples?

**Line 113: we have added the following precision “unpreserved”.**

Line 158, Sarcocystis was not in the multiplex panel?

***Sarcocystis* was not present in the multiplex panel. The isolate analyzed by Sanger sequencing was diagnosed by microscopy.**

Line 197, Microscopy, PCR? Presumably combined please state.

**Line 211: we have added the following precision “by microscopy and/or PCR”.**

Line 236, *Strongyloides stercoralis* is a species, what do the authors mean by species identification failed in that case?

**The initial sentence caused confusion:** “*Strongyloides stercoralis* (n=4), *Dicrocoelium* sp. (n=2), and *Schistosoma* sp. (n=2) were also detected but their species identification failed.” **Species identification was performed only on the two *Schistosoma* strains. We failed to confirm these two strains with our in-house sequencing PCR. However,** **the AMPLIQUICK® Helminths assay targets *Schistosoma mansoni*** **according to the manufacturer’s instructions*.* So, line 251, we have added “*mansoni*” and removed the confusing end of the sentence “but their species identification failed”.**

Line 260 and further, the authors seem to ignore the accumulating evidence that *Blastocystis* and *Dientamoeba* *fragilis* are indicators for a healthy microbiome rather than causing disease. This should at least be discussed.

**We thank reviewer 1 for this relevant comment, and we agree with him. We have added two recent analyses that supports this point: Piperni *et al*. Intestinal *Blastocystis* is linked to healthier diets and more favorable cardiometabolic outcomes in 56,989 individuals from 32 countries. Cell. 2024.; Muhsin-Sharafaldine *et al*. *Dientamoeba fragilis* associated with microbiome diversity changes in acute gastroenteritis patients. Parasitol Int. 2023.**

**Lines 321-325: we have discussed this point as follows: “Even though the presence of *Blastocystis* sp. and *D. fragilis* was reported in individuals with intestinal inflammation or irritable bowel syndrome, recent studies associated them to healthier microbiomes, suggesting a beneficial effect (23-28).”**

Line 380, rephrase intestinal parasitic infections

**Line 444: we have made the correction.**

Reviewer #2:

I would like to thank the editor who offered me this evaluation work.

Since I am not a native English speaker, my proposed changes will only concern the work presented and not the grammar or syntax.

In this manuscript, the authors aimed to provide an epidemiological update on intestinal parasites found in community care in France and Luxembourg.

The paper is clear and well written. This study is of interest because there is limited data in the literature about the prevalence of intestinal parasite in these two countries.

**We thank reviewer 2 for these nice comments regarding our study.**

Major comment:

One major comment concerns the lack of data on diagnostic methods and the absence of exploitation of Microscopy/ PCR data.

**We thank reviewer 2 for this relevant comment, and we obviously agree with him because we are also very interested by that issue. However, the exploitation of microscopy versus PCR data and performance evaluation of the AMPLIQUICK® pretreatment and amplification assays was not an objective of this study which aimed to provide updated data about parasite prevalence in our area.**

**The assessment of these tools was performed by colleagues (Argy *et al.* in Prep, Costa *et al*. in Prep). Adding and discussing these data could be confusing with the main objective of our study. However, below you will find the data and analyses that have been requested as supplementary data. In the main manuscript, an additional evaluation of the microscopy/PCR data was incremented to determine if repetition of sampling in molecular analysis, as in microscopy, can increase sensitivity.**

In the material and method section, the authors present no data on the articulation between the two types of diagnostic methods based on either microscopy or PCR. How were mismatches between the 2 types of test handled (patient PCR(+)/ microscopy(-) or vice versa)? Did one technique correct the results of the other?

The flow chart doesn't provide any information on this subject, and seems to put the emphasis on PCR, since samples with an invalid PCR control were excluded from the study, whereas microscopy should have been available.

**Lines 120-121: we have added the following precision “Patients were considered positive if at least one (or both) of the two diagnostic methods used was positive.”**

**Line 211: we have added the following precision “by microscopy and/or PCR”.**

**Study flowchart (Figure 1) has been updated in this regard.**

Even if this is an epidemiological study, the proportion of each diagnostic method (PCR or microscopy) that enabled detection of the various parasites must be clearly shown for each microorganism. In the same way as the authors investigated the influence of the variables gender, region, season or age on positive patients, I believe it is necessary to do the same with the variables "use of microscopic diagnosis" or "use of PCR diagnosis".

**We have added this analysis in Supplementary Table 6. Lines 295-296: we have added the following precision “The parasite prevalence according to the diagnostic method used is summarized in Supplementary Table 6”. We discussed these results lines 427-429 by adding the following precision “The use of PCR assays increases the proportion of positive patients and strengthens the statistical significance for some variables (e.g., *Giardia* and region or *Cryptosporidium* and season).”**

Given that each stool was analyzed by PCR and microscopy, a comparison of the number of positives obtained by the two types of method would make it possible to identify the gain in detection linked to PCR, as suggested by the authors in line 80 ("The use of molecular assays shows that intestinal parasites are underestimated in industrialized countries"). This has not been done and is missing from the study.

Finally, a comparison between PCR and microscopy could give an initial idea of the performance of the Biosynex test, for which no publication exists to date.

**We have addressed this issue in a specific paragraph lines 255-271:**

**“Comparison between PCR and microscopy.**

**A comparison of the number of positive patients obtained by the two types of methods was summarized in Fig 4 and Supplementary Table 2. We found that more than half of the cases were diagnosed by PCR alone (blank bars, Fig 4), indicating the higher sensitivity of molecular methods. Some others can only be diagnosed by microscopy (dark bars, Fig 4), or were missed by PCR (see *Blastocystis* sp. and *E. vermicularis*). Parasite prevalence according to the diagnostic method used and the number of stool samples per patient was summarized in Supplementary Table 3. The overall prevalence was significantly increased with the number of stools collected per patient by microscopy and PCR combined, as well as by microscopy alone or PCR alone (p=0.001, p<0.001 and p=0.003, respectively). These results were similar when considering the overall prevalence excluding *Blastocystis* sp. (p=0.02, p=0.02 and p=0.045, respectively), or specifically considering *Blastocystis* sp. (p=0.002, p<0.001 and p=0.03, respectively). Parasite carriage was then assessed in the subset of positive patients with 3 stool samples. More than half of the patients were diagnosed with parasites in only one or two of the three samples, both overall and by parasite (Fig 5). Looking at the 21 patients with co-occurrences in this subset, a second or third sample was often needed to detect all the parasites carried by a patient (Supplementary Table 4).”**

**We have discussed these results lines 425-433:**

**“Finally, a comparison between PCR and microscopy, although this study was not designed for it, shows some preliminary data that will be interesting to develop in future studies. The use of PCR diagnostics increases the proportion of positive patients and strengthens the statistical significance for some variables (e.g., *Giardia* and region or *Cryptosporidium* and season). The prevalence increases significantly with the multiplication of samples by microscopy as well as by PCR, but not for all parasites. In positive patients with multiple stool samples, not all parasites were always detected in each sample, even with the PCR method. Thus the increased sensitivity of molecular assays does not make it possible to avoid repeated sampling for parasitological testing.”**

Minor comments:

1)	Since Microsporidia belong to the fungi kingdom, it is theoretically necessary to add microsporidia infections whenever the term "intestinal parasite" or "intestinal parasitic infection" is used. In the title, add "microsporidiosis": “Current status of intestinal parasitosis and microsporidiosis…”

For the rest of the text, it's a bit cumbersome to systematically add the term "microsporidia", but it should be reiterated at the start of each important new section, in the conclusions and in the abstract.

**Lines 1, 46, 72, 87, 212, 281, 302, 308: we have added the following precision “microsporidiosis”.**

2)	Line 78: Define the term "pathogenic" in materials and methods. Authors should identify by name the species they have considered to be pathogenic.

**Lines 165-170: we have added the following section in materials and methods “Definition of pathogenic or non-pathogenic parasite. The presence of parasites for which the pathogenic character is still debated was reported as carriage and were the following: *Blastocystis* sp*., D. fragilis*, *Entamoeba coli*, *Endolimax nana*, *Entamoeba dispar*, *Entamoeba hartmanni*, *Iodamoeba butschlii* and *Chilomastix mesnilii*. All other parasites found in this study were considered pathogenic and their presence was reported as an infection.**

3)	Line 91: Add the Calderao et al study from Italy (Calderaro et al. Intestinal parasitoses in a tertiary-care hospital located in a non-endemic setting during 2006-2010. BMC Infect Dis. 2014, doi: 10.1186/1471-2334-14-264. PMID: 24886502)

**Line 93: we have added this study.**

4)	Line 112-113: Stool samples were collected prospectively from all regions of France and Luxembourg. No description of pre-analytical conditions was provided by the authors (storage, preservation solution, time to microscopic analysis). Add these information in the material and methods section.

**Line 113: we have added the following precision “unpreserved”.**

**Lines 115-117: we have added the following precision “The samples from primary care prescriptions were transported and stored at 4°C for two to five days prior to testing for the presence of ova, cysts, larvae, vegetative forms, adult stage helminths, and parasite and microsporidia DNA.”**

5)	Line 114 -115: Add “adult-stage helminths” and “parasite and microsporidia DNA” to the sentence “were investigated for the presence of ova, cysts, larvae, and vegetative forms.

**Line 117: we have made the correction.**

6)	Line 139: According to the manufacturer’s instructions, the AMPLIQUICK® Protozoans assay targets *Encephalitozoon* spp. (not *Encephalitozoon intestinalis*).

**Line 144: we have made the correction.**

7)	Line 142-143: According to the manufacturer’s instructions, the AMPLIQUICK® Helminths assay targets *Diphyllobothrium* *latum* and *Schistosoma mansoni* (not *Diphyllobothrium* sp. and *Schistosoma* sp.)

**Lines 147-148: we have made the correction.**

8)	Line 206: Results are presented per patient. The same patient may therefore have several stools. The authors do not describe how they considered a patient with different parasites isolated from different stool samples (coinfections? independent parasites?). Add these information in the material and methods section.

**Lines 121-123: we have added the following precision “Results are presented per patient: different stool sample results for each patient were pooled for analysis (i.e., reported as co-occurrence if different parasites were found in different samples).”**

9)	Line 211: It's a pity that the authors didn't try to associate parasitic co-occurrences with the data collected (gender, region, season or age, method of diagnosis).

**We have added this analysis in Supplementary Table 1. Lines 420-422: we have added the following precision “The age group 5-14 years was significantly more co-infected than others (Supplementary Table 1). This is explained by the main coinfection, *Blastocystis* sp./*D. fragilis*, which show the same difference in age class repartition for each of them.” Another difference for co-occurrences was found for sex distribution. Lines 232-234: we have added the following precision “Significantly more males than females were co-infected (respectively 13.4% vs 7.3%, p<0.001, Supplementary Table 1).”**

10)	Line 240-247: It is also a pity that a multivariate analysis was not carried out to identify the parameters that best explain the "Parasite detection" variable. For example, for *Giardia*, it would be interesting to analyze the explanatory variables "Region (p=0.04)", "Season (p=0.06)" or "Age 5-14" to identify which of these parameters best explains positivity for this parasite. The same can be done for any other parasites detected.

**The study of factors associated with parasitic carriage was exploratory, therefore multivariable analyses were not planned in the statistical analysis plan. To perform multivariable analyses, we have to (i) focus on *Blastocystis* sp. and *Dientamoeba* *fragilis* because there are not enough infections for the other parasites; and (ii) add sex, region, season, and age as covariates, because there are all clinically relevant. We have added this analysis in Supplementary Table 5. Multivariable analysis confirmed that *D. fragilis* was more prevalent in winter (OR=0.69 (0.50; 0.96), p=0.03) and that *D. fragilis* was significantly more prevalent in children aged 5-14 years old (OR=4.17 (2.32; 7.49), p<0.001), while *Blastocystis* sp. was less prevalent in children <5 years old (OR>2.27 (1.05; 4.90), p<0.04).**

**Lines 187-189: we have added the following precision** “**Multivariable analyses were performed for the two most prevalent parasites (*Blastocystis* sp. and *D. fragilis*) using logistic regressions and the following independent variables: sex, region, season, and age. The results are expressed as odds ratio and 95%CI.”**

**Lines 293-295: we have added the following precision “Multivariable analysis confirmed the differences observed for *Blastocystis* sp. and *D. fragilis* (Supplementary Table 5).”**

11)	Line 242: The 2 prevalence values have been reversed. Replace « 1.3% vs 2.8% in the North » with « 2.8% vs 1.3% in the North».

**Line 284: we have made the correction.**

12)	Line 263: The sentence "The aim of this study was to make an epidemiological update of intestinal parasitosis in Europe..." needs to be reworded. The term "Europe" is geographically too broad for an epidemiological study carried out in only 2 neighboring countries (one of which has a small surface area).

**Line 310: We have rephrased it as follows: “The aim of this study was to make an epidemiological update of intestinal parasitosis in these two countries.”**

13)	Line 272: To the sentence “a drawback that reinforces the need to conserve microscopic skills” could be added “or to push the manufacturers to develop new PCR for these targets”

**Lines 319-320: we have added the suggestion.**

14)	Line 295: Add the Tchamwa Bamini et al. study from France (Prevalence of *D. fragilis*: 6.5%)

(Tchamwa Bamini et al. No evidence of pathogenicity of *Dientamoeba fragilis* following detection in stools: A case-control study. Parasite. 2024;31:40. doi: 10.1051/parasite/2024041. Epub 2024 Jul 24. PMID: 39052010)

**Lines 345-347: we have added this study as follows: “In France, data are scarce but three recent molecular prospective studies reported a prevalence of 2.3% in 488 patients, 11.9% from 588 samples (for 350 patients) and 6.5% in 673 patients”.**

15)	Line 297: The significantly higher prevalence in children under the age of 14 observed in this study is in line with the results of the Tchamwa Bamini et al. study.

**Lines 348-350: we have added the suggestion.**

16)	Line 344: It would be interesting to know how *Enterobius vermicularis* cases were diagnosed (PCR or microscopy).

**Lines 397-398: we have added the following precision: “Five cases were diagnosed by microscopy, the others by PCR only (Supplementary Figure 1).”**

17)	Line 372 (and line 66 and 81): I think the sentence “The high prevalence of intestinal parasites underlines the importance of investigating gastrointestinal disorders through parasite examination, even in developed countries” needs to be amended. Indeed, almost four-fifths of identified parasites have no proven pathogenicity, and this sentence makes a clumsy shortcut between this very high overall prevalence and the need for diagnosis linked to the presence of gastrointestinal disorders. For greater clarity, I suggest: "The high prevalence of pathogenic intestinal parasites (about 7%) underlines the importance of investigating gastrointestinal disorders through parasite examination, even in developed countries".

**Lines 66, 82, 322, 437: we have made the correction.**
