# Supplementary material for: Current status of intestinal parasitosis and microsporidiosis in industrialized countries: Results from a prospective study in France and Luxembourg
Source: PLoS Negl Trop Dis. 2024 Dec 23;18(12):e0012752. doi: 10.1371/journal.pntd.0012752 (PMC11706478; doi:10.1371/journal.pntd.0012752)
Supplement: Attachment — Submitted filename: PNTD_revised_highlights_PP_CN_rev.docx [file pntd.0012752.s009.docx]

**Full Title**: Current status of intestinal parasitosis and microsporidiosis in industrialized countries: results from a prospective study in France and Luxembourg.

**Short Title**: Current status of intestinal parasitosis in industrialized countries.

**Authors:** Maxime Moniot^1,2^ (https://orcid.org/0000-0002-1471-6945), Céline Nourrisson^1,2,3^ (https://orcid.org/0000-0002-4003-805X), Eloïse Bailly^4,5^, Céline Lambert^6^, Patricia Combes^2^, Philippe Poirier^1,2,3^ (https://orcid.org/0000-0001-9173-2634).

**Affiliations**

^1^ Service de Parasitologie-Mycologie, CHU Clermont-Ferrand, 3IHP, Clermont-Ferrand, France.

^2^ Centre National de Référence Cryptosporidioses, Microsporidies et autres protozooses digestives (Laboratoire associé), CHU Clermont-Ferrand, Clermont-Ferrand, France.

^3^ Microbes, Intestin, Inflammation et Susceptibilité de l'Hôte (M2iSH), UMR Inserm/ Université Clermont Auvergne U1071, USC INRA 2018, France.

^4^ Laboratoire de Parasitologie-Mycologie, CHU Dijon Bourgogne, Dijon, France.

^5^ Centre National de Référence Cryptosporidioses, Microsporidies et autres protozooses digestives (Laboratoire associé), CHU Dijon Bourgogne, Dijon, France.

^6^ Unité de Biostatistiques, DRCI, CHU Clermont-Ferrand, Clermont-Ferrand, France.

**Corresponding author:**

E-Mail: [ppoirier@chu-clermontferrand.fr](mailto:ppoirier@chu-clermontferrand.fr) (PP)

**Keywords:** intestinal parasitosis, nationwide epidemiology, *Blastocystis* sp., *Dientamoeba fragilis*, *Cryptosporidium* spp*.*, *Giardia intestinalis*, *Enterobius vermicularis*, molecular diagnosis, microscopic diagnosis

**Statement**

**Ethical statement:** The study was approved by local Ethics Committee under number 2023-CF103 (IRB00013412, “CHU de Clermont-Ferrand IRB #1”). Due to the anonymity of the study, formal consent was not obtained.

**Funding statement:** This work was supported by internal laboratory funding. This research did not receive any specific grant from funding agencies in the public, commercial, or not-for-profit sectors.

**Data availability:** The datasets generated during and/or analyzed during the current study are available from the corresponding author on reasonable request.

**Conflict of interest statement:** None.

**Authors’ contributions:** PP, CN and MM conceived and designed the study. MM collected data, did the literature search and drafted the manuscript. MM, PC end EB did sequencing experimentations. CL did statistical analysis. CN, CL, PC, EB and PP edited the manuscript. PP conceived the study and oversaw the manuscript. All authors read and approved the final manuscript.

**Words count:** 4188 (abstract 251)

**Figures and Tables:** 7

**Abstract**

**Background.**

Human intestinal parasitosis and microsporidiosis are a global health concern, mostly in endemic areas but should not be neglected elsewhere. Recent nationwide epidemiological data are scarce, especially from community based care and developed countries. Diagnosis by molecular tools are increasing and several commercial gastrointestinal panel assays including protozoans and/or helminths are now available. These news tools improve the knowledge into real human parasite epidemiology. This study provides an epidemiological update on intestinal parasites found in community care in France and Luxembourg.

**Methodology/Principal Findings.**

Two thousand fifty-six stools from primary care patients were analyzed for the presence of intestinal parasites (IPs) during two different seasons of 2022, the winter and the summer, corresponding to more than 1500 patients from all over France and Luxembourg. Parasite detection was performed combining standard microscopy (merthiolate-iodine-formaldehyde and Bailenger concentration procedures) with two molecular panel assays (AMPLIQUICK Fecal Pretreatment, AMPLIQUICK Protozoans and AMPLIQUICK Helminths, BIOSYNEX, France). The prevalence of IPs in primary care patients reached 33.2%. *Blastocystis* sp. and *Dientamoeba fragilis* were the most frequently detected parasites in 20.5% and 13.1% of patients, respectively. Co-occurrence with two or more parasites was detected in 9.9% of patients. For some parasites, patterns according to gender, age, geography or season have been observed.

**Conclusion/Significance.**

The high prevalence of pathogenic IPs (about 7%) underlines the importance of investigating gastrointestinal disorders through parasite examination, even in developed countries. Parasites molecular characterization give new insights and should encourage further research as industrialized countries are not exempt of parasitic circulation and a better survey is necessary.

## Author Summary

Recent nationwide epidemiological data on human intestinal microsporidiosis and parasites are scarce, especially among community-based care patients and in developed countries. Using novel data, we present an exhaustive epidemiology of intestinal parasites (IPs)in France and Luxembourg in 2022 from the community-based care patients. In this study, a high prevalence of IPs of 33.2% was found. *Blastocystis* sp. and *Dientamoeba* *fragilis* were the most frequently detected parasites, in 20.5% and 13.1% of patients, respectively. Considering only pathogenic parasites, the prevalence was 7.0%. Differences according to sex, region, season or age group were observed. Co-occurrence of two or more parasites was observed in 9.9% of patients. The use of molecular assays shows that IPs are underestimated in industrialized countries. The high prevalence of pathogenic IPs underlines the importance of investigating gastrointestinal disorders through parasite examination. The detection of parasites, pathogenic or not, remains a marker of the faecal-oral route of transmission and results should be interpreted accordingly.

## Introduction

Human intestinal parasitosis and microsporidiosis are a global health concern, mostly in endemic areas such as tropical countries, but should not be neglected elsewhere (1). Recent nationwide epidemiological data are scarce in Europe, especially from community-based care patients. In France and Luxembourg, intestinal parasitosis surveys are not mandatory and so there is little epidemiological information, and the incidence of the infection is considered to be low. Most studies to date have centred on either extensive parasite research in a single centre (2–4), or parasitic research in a wider geographic area (multicentre studies) but focusing on specific parasites (5,6). Another limitation is the population studied, which is often a restricted category like children, inpatients or travellers, for example (2,3,7). Epidemiological studies of outpatients are therefore needed to better estimate the prevalence of intestinal parasites (IPs) on a large scale and to understand parasite circulation in the general population.

The recent development of several commercial multiplex PCR panel assays and their prospective assessment have provided greater data on parasitic infections. These tools are more expensive than microscopy but can be used in routine laboratory practice and improve the sensitivity of targeted parasites (8–10). However, the relevant studies suffer from the same limitations concerning population and certain parasite targets are still lacking for exhaustive results. In the present study, we performed an epidemiological update on IPs circulating in community care in France and Luxembourg in 2022 from both winter and summer sample collections. We included patients without any *a priori* selection for whom a consultation in the primary care setting had led to a prescription of a parasitological test. We combined standard microscopic methods with molecular assays to obtain exhaustive epidemiological data and performed further molecular analyses for certain parasites such as species identification and genotyping.

## Materials and methods

**Clinical samples.**

Unpreserved stool specimens were prospectively collected during two periods in 2022, in the winter from February 13^th^ to March 25^th^, and in summer from the 1^st^ to the 30^th^ of August. The samples from primary care prescriptions were transported and stored at 4°C for two to five days prior to testing for the presence of ova, cysts, larvae, vegetative forms, adult stage helminths, and parasite and microsporidia DNA. The sample was accompanied by patient data, including sex, age and ZIP code of residence. Three patients for whom analyses were performed in both sampling periods were considered as two different patients because several months had elapsed between the two periods. Patients were considered positive if at least one (or both) of the two diagnostic methods used was positive. Results are presented per patient: different stool sample results for each patient were pooled for analysis (i.e., reported as co-occurrence if different parasites were found in different samples).

**Macroscopic and microscopic examinations.**

All samples were prospectively analysed by experienced operators in the Laboratory of Parasitology of the University Hospital of Clermont-Ferrand, France. For each sample, a macroscopic examination was performed to identify adult-stage helminths, followed by microscopic examination of fresh homogenized stools, and of merthiolate-iodine-formaldehyde (MIF) and Bailenger concentration techniques. In parallel to these analyses, each stool was prospectively aliquoted and kept at -20°C until use for retrospective molecular analysis.

**Molecular panel assays**.

At the end of patient inclusion, frozen samples were mechanically and chemically pretreated using the AMPLIQUICK Faecal Pretreatment assay (BIOSYNEX, France). Briefly, 200 mg of stool were ground with manufacturer’s beads and then incubated at 95°C in lysis buffer for 5 minutes. DNA extraction of 200 µL of the supernatant was performed with the NucleoMag DNA Microbiome kit (Macherey Nagel, France) according to the manufacturer’s instructions. The two molecular assays AMPLIQUICK Protozoans and AMPLIQUICK Helminths (BIOSYNEX, France) were used for the amplification of 20 different parasites, 10 protozoa and 10 helminths. Each panel included an internal control (to detect PCR inhibitors) and an in-process control (to validate the extraction and purification steps). All samples that did not satisfy each assay control from each panel were excluded from the analysis. The AMPLIQUICK Protozoans assay targets *Blastocystis* sp., *Cryptosporidium* sp., *Cyclospora cayetanensis*, *Cystoisospora belli*, *Dientamoeba fragilis*, *Encephalitozoon* spp*.*, *Entamoeba dispar*, *Entamoeba histolytica*, *Enterocytozoon bieneusi* and *Giardia intestinalis*. The AMPLIQUICK Helminths assay targets *Ancylostoma duodenale*, *Ascaris lumbricoides*, *Diphyllobothrium* *latum*, *Enterobius vermicularis*, *Hymenolepis nana*, *Necator americanus*, *Schistosoma* *mansoni*, *Strongyloides stercoralis*, *Taenia* sp. and *Trichuris trichiura*.

**Genomic analyses.**

To obtain full epidemiological data, species identification or genotyping was performed when the amount of DNA remaining was sufficient. All *Blastocystis* sp. positive samples were subtyped as previously described by targeting the small-subunit (SSU) ribosomal ribonucleic acid (rRNA) gene (11). *Dientamoeba* *fragilis* genotypes 1 and 2 were distinguished by sequencing the SSU rRNA gene (12). Species identification of *Cryptosporidium* was performed by an in-house PCR targeting a 258-bp DNA fragment located in the SSU rRNA gene (13). Genotyping of *Cryptosporidium* (*i.e*. identification of subtype families and subtypes) was performed by next-generation sequencing of the highly polymorphic gp60 encoding gene as previously described (14). *Giardia intestinalis* assemblages were determined by Sanger sequencing of the SSU rRNA gene as previously described (15). Species identification of *Taenia* sp., *Hymenolepis* sp. and *Schistosoma* sp. were performed by sequencing the mitochondrial cytochrome c oxidase subunit 1 (COX1) gene (16). *Encephalitozoon* species identification and *Enterocytozoon* *bieneusi* genotyping were performed by sequencing the internal transcribed spacer (ITS) region (17,18). Finally, COX1 gene was sequenced to achieve species identification of *Sarcocystis* sp. (19).

**Definition of pathogenic or non-pathogenic parasite.**

The presence of parasites for which the pathogenic character is still debated was reported as carriage and were the following: *Blastocystis* sp., *D. fragilis*, *Entamoeba coli*, *Endolimax nana*, *Entamoeba dispar, Entamoeba hartmanni, Iodamoeba butschlii* and *Chilomastix mesnilii.* All other parasites found in this study were considered pathogenic and their presence was reported as an infection.

**Statistical analysis.**

This cross-sectional study, conducted on a sample population, should make it possible to generalize the results to the entire target population. A margin of error on the estimate is defined to calculate the number of patients required in addition to the expected proportion of the primary outcome. Thus, for an expected proportion of intestinal parasitic carriage of about 50% (proportion requiring the highest number of patients), the inclusion of at least 1068 patients would make it possible to obtain an accuracy of ±3% of this proportion. Statistical analysis was performed with Stata software (version 15; StataCorp, College Station, Texas, USA). All tests were two-sided, with an alpha level set at 5%. No correction for multiple testing was applied in the secondary analyses (20). Categorical data are presented as numbers and percentages, and age as mean ± standard deviation. The overall prevalence of intestinal parasitic carriage, and the prevalence of each parasite, were presented with a 95% confidence interval (95%CI), estimated by a binomial distribution. Factors associated with parasitic carriage (sex, geographical origin, season of sampling, and age) were studied with the Chi-squared test or the Fisher’s exact test. For comparisons according to age groups, a post hoc multiple comparisons test (Marascuilo) was performed if omnibus p-value was less than 0.05. Multivariable analyses were performed for the two most prevalent parasites (*Blastocystis* sp. and *D. fragilis*) using logistic regressions and the following independent variables: sex, region, season, and age. The results are expressed as odds ratio and 95%CI.

## Results

**Study population.**

Overall, 2056 stool samples taken from 1589 patients were analysed during the winter and summer periods (Fig 1). Twenty-two stools were excluded because of at least one invalid PCR control, leaving 2034 stools sampled from 1570 patients, made up of 1072 stools (840 patients) sampled in the winter and 962 (730 patients) in the summer. The 294 stools (283 patients) from Luxembourg were collected only during the winter period. Regarding seasonality, there was no difference between winter and summer in terms of age (42.7 ± 24.5 *vs* 44.9 ± 25.0 years old, respectively, p=0.08) or sex ratio (male: 42.6% *vs* 42.9%, p=0.92). The geographical origin of the samples showed that more were collected from the North than the South, 1246 stools (983 patients) vs 781 (582 patients), respectively (Fig 2A). Ages ranged from one month to 99 years in a population comprising 276 children (*i.e.* <18 years old) and 1293 adults (one birth date was lacking) with a sex ratio male/female of 0.75 (n=899 females, 57.3%, n=671 males, 42.7%) (Fig 2B).

**Fig 1.** **Study flowchart.**

**Fig 2.** **Study population.** (A) The patient’s place of residence at time of sampling is represented on a distribution map downloaded from Servier Medical Art (<https://smart.servier.com/citation-sharing/>) (n=1565, five ZIP code lacking). The grey line represents separation between North and South areas for statistical analysis. (B) The distribution of patients by sex and age is represented on the corresponding population pyramid (n=1569).

**Parasites detected.**

All parasites detected in this study by microscopy and/or PCR are listed in Fig 3. The overall prevalence of intestinal microsporidiosis and parasitic carriage was 33.2% (n=521/1570 patients, 95%CI: 30.9 to 35.6). *Blastocystis* sp. and *Dientamoeba* *fragilis* were the most frequently detected parasites being identified in 20.5% (n=322) and 13.1% (n=206) of patients, respectively, followed by *Giardia intestinalis* (1.9%, n=30), *Cryptosporidium* spp. (1.9%, n=30), *Enterobius vermicularis* (1.9%, n=29), and amoeba-like *Entamoeba coli* (1.5%, n=23), *Endolimax nana* (1.2%, n=19) and *Entamoeba dispar* (1.1%, n=17). No *Entamoeba histolytica* was detected. Some other protozoa were found such as *Cystoisospora belli* (n=3), *Entamoeba hartmanni* (n=2), *Iodamoeba butschlii* (n=2), *Chilomastix mesnili* (n=1), *Sarcocystis* sp. (n=1) and *Cyclospora cayetanensis* (n=1).

**Fig 3. Prevalence by parasite with coinfections distribution.** Results are presented by patient (n=1570). Parasites are sorted from most to least prevalent. Dark blue boxes indicate cases with only one parasite detected, the others indicate cases with two or more parasites detected. Coinfections appear vertically with dotted lines to make reading easier. Parasites not shown are equal to zero. CI: confidence interval.

Coinfection with two or more parasites occurred in 156 patients (9.9%, 95%CI: 8.5 to 11.5), of whom 126 (8.0%) were coinfected with two parasites, 25 (1.6%) with three parasites, 4 (0.3%) with four parasites, and one (0.1%) with five parasites. This last patient was coinfected with *D. fragilis*, *G. intestinalis, E. coli*, *Trichuris trichiura*, and *Hymenolepis* *nana*. The most frequent associations between two parasites were *Blastocystis* sp./*D. fragilis* (n=77), followed by *D. fragilis*/*E. vermicularis* (n=18), *Blastocystis* sp./*G. intestinalis* (n=15), *Blastocystis* sp./*E. nana* (n=12) and *Blastocystis* sp./*E. coli* (n=11) (Fig 3). Significantly more males than females were co-infected (respectively, 13.4% vs 7.3%, p<0.001, S1 Table).

Molecular analysis up to the species or genotype level was performed for the parasites listed below. However, not all were successful for a variety of reasons, including insufficient amount of DNA, degraded DNA or insufficient amplification of the target DNA for sequencing analysis. Of the 322 patients positive for *Blastocystis* sp., 252 isolates were successfully subtyped as follows, from most to least prevalent: ST3 (25.8%), ST4 (25.0%), ST2 (22.2%), ST1 (15.1%), ST7 (7.1%), ST6 (3.6%), and ST8 (1.2%). Likewise, of the 206 patients positive for *D. fragilis*, 143 isolates were successfully genotyped and shown to be mainly of genotype 1 (97.9%). Only three patients (2.1%) were detected with genotype 2. Of the 30 patients positive for *G. intestinalis,* 16 isolates were successfully genotyped into 6 assemblages A, and 10 assemblages B. Among the 30 patients positive for *Cryptosporidium* sp., 14 *Cryptosporidium parvum* and 2 *Cryptosporidium hominis* were identified. The following *C. parvum* subtypes were identified: IIaA15G2R1 (n=10), IIaA14R1 (n=1), IIaA17G2R1 (n=1), IIaA18G3R1 (n=1), and IIaA22G1 (n=1). For *C. hominis*, the two genotypes found were IbA9G3 and IbA13G3. In the seven patients harbouring microsporidia, the species involved were *Enterocytozoon bieneusi* (n=6) and *Encephalitozoon* sp. (n=1). Unfortunately, *Encephalitozoon* species identification failed but successful genotyping of four *E. bieneusi* isolates showed the presence of genotypes A (n=1), Peru11 (n=1), PigEbITS3 (n=1), and Wildboar3 (n=1). Of the five positive cases of *Taenia* sp., four were successfully identified as *T. saginata*. The *Sarcocystis* isolate was identified as *Sarcocystis hominis*. *Strongyloides stercoralis* (n=4), *Dicrocoelium* sp. (n=2), and *Schistosoma* *mansoni* (n=2) were also detected.

**Comparison between PCR and microscopy**

A comparison of the number of positive patients obtained by the two types of methods was summarized in Fig 4 and S2 Table. We found that more than half of the cases were diagnosed by PCR alone (blank bars, Fig 4), indicating the higher sensitivity of molecular methods. Some others cases can only be diagnosed by microscopy (dark bars, Fig 4), or were missed by PCR (see *Blastocystis* sp. and *E. vermicularis*). Parasite prevalence according to the diagnostic method used and the number of stool samples per patient was summarized in S3 Table. The overall prevalence was significantly increased with the number of stools collected per patient by microscopy and PCR combined, as well as by microscopy alone or PCR alone (p=0.001, p<0.001 and p=0.003, respectively). These results were similar when considering the overall prevalence excluding *Blastocystis* sp. (p=0.02, p=0.02 and p=0.045, respectively), or specifically considering *Blastocystis* sp. (p=0.002, p<0.001 and p=0.03, respectively). Parasite carriage was then assessed in the subset of positive patients with 3 stool samples. More than half of the patients were diagnosed with parasites in only one or two of the three samples, both overall and by parasite (Fig 5). Looking at the 21 patients with co-occurrences in this subset, a second or third sample was often needed to detect all the parasites carried by a patient (S4 Table).

**Fig 4. Comparison of the number of positive patients obtained by microscopy and/or PCR.** Histograms show the number of cases diagnosed by PCR alone (blank bars), by microscopy alone (dark bars), or by both methods (gray bars). **Enterocytozoon bieneusi* + *Encephalitozoon* sp.

**Fig 5. Number of positive stool samples in the subset of positive patients with 3 stool samples according to the diagnostic method used.** M: Microscopy, PCR: Polymerase Chain Reaction.

**Factors associated with intestinal parasitic carriage.**

There was no effect of sex, season or geographical area on overall parasite and microsporidiosis prevalence (Table 1). No significant difference in geographical origin was found between individual parasites from the North and the South of the country except for *G. intestinalis*, which was more prevalent in the South (2.8% *vs* 1.3% in the North, p=0.04) (Fig 6). *Cryptosporidium* sp. was more prevalent in the summer (3.0% *vs* 1.0% in the winter, p=0.003), while *Dientamoeba fragilis* was more prevalent in the winter (15.0% *vs* 11.0% in the summer, p=0.02), as was *Entamoeba coli* (2.0% *vs* 0.8% in the summer; p=0.048) (Table 1). *Entamoeba coli* was also more prevalent in males than in females (2.2% *vs* 0.9%, respectively, p=0.03), as was *Entamoeba dispar* (1.9% vs 0.4%, respectively, p=0.005). Regarding age, *D. fragilis* was significantly more prevalent in children aged 5-14 years old (49.6% *vs* less than 19.2% in other age groups, p<0.001), while *Blastocystis* sp. was less prevalent in children <5 years old (8.3% *vs* more than 16.6% in other age groups, p=0.004). *Enterobius vermicularis* was also predominant in the 5- to 14-year-old age class with a prevalence of 7.0%. Multivariable analysis confirmed the differences observed for *Blastocystis* sp. and *D. fragilis* (S5 Table). The parasite prevalence according to the diagnostic method used is summarized in S6 Table. The use of PCR assays increases the proportion of positive patients and strengthens the statistical significance for some variables (e.g., *Giardia* and region or *Cryptosporidium* and season).

**Fig 6. Prevalence of the five most prevalent parasites according to sex, geographical origin, season of sampling and age.** Results are presented as percentage of patients (n=1570 for sex and season, n=1569 for age class, n=1565 for geographical origin). yo: years old.

**Table 1.** **Parasite and microsporidiosis prevalence according to sex, geographical origin, season of sampling and age.**

|  | **Sex** | | |  | **Region** | | |  | **Season** | | |  | **Age (years)** | | | | | |
| --- | --- | --- | --- | --- | --- | --- | --- | --- | --- | --- | --- | --- | --- | --- | --- | --- | --- | --- |
|  | **Female**  **(n=899)** | **Male**  **(n=671)** | **p** |  | **North**  **(n=983)** | **South**  **(n=582)** | **p** |  | **Winter**  **(n=840)** | **Summer**  **(n=730)** | **p** |  | **<5**  **(n=120)** | **5-14**  **(n=115)** | **15-24**  **(n=169)** | **25-44**  **(n=413)** | **≥45**  **(n=752)** | **p** |
| Overall positivity | 287 (31.9) | 234 (34.9) | 0.22 |  | 328 (33.4) | 190 (32.6) | 0.77 |  | 281 (33.5) | 240 (32.9) | 0.81 |  | 37 (30.8) | 70 (60.9) | 51 (30.2) | 134 (32.4) | 229 (30.5) | **<0.001** |
| *Blastocystis* sp*.* | 171 (19.0) | 151 (22.5) | 0.09 |  | 211 (21.5) | 110 (18.9) | 0.23 |  | 171 (20.4) | 151 (20.7) | 0.87 |  | 10 (8.3) | 26 (22.6) | 28 (16.6) | 87 (21.1) | 171 (22.7) | **0.004** |
| *Dientamoeba fragilis* | 111 (12.3) | 95 (14.2) | 0.29 |  | 122 (12.4) | 83 (14.3) | 0.29 |  | 126 (15.0) | 80 (11.0) | **0.02** |  | 23 (19.2) | 57 (49.6) | 18 (10.7) | 45 (10.9) | 63 (8.4) | **<0.001** |
| *Giardia intestinalis* | 15 (1.7) | 15 (2.2) | 0.46 |  | 13 (1.3) | 16 (2.8) | **0.04** |  | 11 (1.3) | 19 (2.6) | 0.06 |  | 1 (0.8) | 5 (4.4) | 3 (1.8) | 6 (1.5) | 15 (2.0) | 0.36 |
| *Cryptosporidium* sp*.* | 14 (1.6) | 16 (2.4) | 0.24 |  | 18 (1.8) | 12 (2.1) | 0.75 |  | 8 (1.0) | 22 (3.0) | **0.003** |  | 3 (2.5) | 4 (3.5) | 4 (2.4) | 5 (1.2) | 14 (1.9) | 0.44 |
| *Enterobius vermicularis* | 16 (1.8) | 13 (1.9) | 0.82 |  | 18 (1.8) | 11 (1.9) | 0.93 |  | 19 (2.3) | 10 (1.4) | 0.19 |  | 4 (3.3) | 8 (7.0) | 6 (3.6) | 5 (1.2) | 6 (0.8) | **<0.001** |
| *Entamoeba coli* | 8 (0.9) | 15 (2.2) | **0.03** |  | 18 (1.8) | 5 (0.9) | 0.12 |  | 17 (2.0) | 6 (0.8) | **0.048** |  | 0 (0.0) | 3 (2.6) | 1 (0.6) | 8 (1.9) | 11 (1.5) | 0.37 |
| *Endolimax nana* | 9 (1.0) | 10 (1.5) | 0.38 |  | 9 (0.9) | 10 (1.7) | 0.16 |  | 7 (0.8) | 12 (1.6) | 0.14 |  | 0 (0.0) | 1 (0.9) | 2 (1.2) | 9 (2.2) | 7 (0.9) | 0.30 |
| *Entamoeba dispar* | 4 (0.4) | 13 (1.9) | **0.005** |  | 10 (1.0) | 7 (1.2) | 0.73 |  | 9 (1.1) | 8 (1.1) | 0.96 |  | 0 (0.0) | 0 (0.0) | 3 (1.8) | 8 (1.9) | 6 (0.8) | 0.18 |
| Microsporidia*** | 4 (0.4) | 3 (0.4) | 1.00 |  | 6 (0.6) | 0 (0.0) | 0.09 |  | 3 (0.4) | 4 (0.5) | 0.71 |  | 1 (0.8) | 0 (0.0) | 1 (0.6) | 1 (0.2) | 4 (0.5) | 0.72 |
| *Taenia* sp*.* | 2 (0.2) | 3 (0.4) | n.d. |  | 4 (0.4) | 1 (0.2) | n.d. |  | 4 (0.5) | 1 (0.1) | n.d. |  | 0 (0.0) | 0 (0.0) | 1 (0.6) | 2 (0.5) | 2 (0.3) | n.d. |
| *Strongyloides stercoralis* | 1 (0.1) | 3 (0.4) | n.d. |  | 2 (0.2) | 2 (0.3) | n.d. |  | 3 (0.4) | 1 (0.1) | n.d. |  | 0 (0.0) | 0 (0.0) | 0 (0.0) | 2 (0.5) | 2 (0.3) | n.d. |
| *Cystoisospora belli* | 2 (0.2) | 1 (0.1) | n.d. |  | 2 (0.2) | 1 (0.2) | n.d. |  | 1 (0.1) | 2 (0.3) | n.d. |  | 0 (0.0) | 0 (0.0) | 1 (0.6) | 0 (0.0) | 2 (0.3) | n.d. |
| *Dicrocoelium* sp*.* | 2 (0.2) | 0 (0.0) | n.d. |  | 2 (0.2) | 0 (0.0) | n.d. |  | 1 (0.1) | 1 (0.1) | n.d. |  | 0 (0.0) | 0 (0.0) | 0 (0.0) | 1 (0.2) | 1 (0.1) | n.d. |
| *Entamoeba hartmanni* | 0 (0.0) | 2 (0.3) | n.d. |  | 0 (0.0) | 2 (0.3) | n.d. |  | 0 (0.0) | 2 (0.3) | n.d. |  | 0 (0.0) | 0 (0.0) | 0 (0.0) | 0 (0.0) | 2 (0.3) | n.d. |
| *Iodamoeba butschlii* | 1 (0.1) | 1 (0.1) | n.d. |  | 1 (0.1) | 1 (0.2) | n.d. |  | 0 (0.0) | 2 (0.3) | n.d. |  | 0 (0.0) | 0 (0.0) | 1 (0.6) | 1 (0.2) | 0 (0.0) | n.d. |
| *Schistosoma* sp*.* | 2 (0.2) | 0 (0.0) | n.d. |  | 1 (0.1) | 1 (0.2) | n.d. |  | 1 (0.1) | 1 (0.1) | n.d. |  | 0 (0.0) | 0 (0.0) | 2 (1.2) | 0 (0.0) | 0 (0.0) | n.d. |
| *Chilomastix mesnili* | 0 (0.0) | 1 (0.1) | n.d. |  | 1 (0.1) | 0 (0.0) | n.d. |  | 1 (0.1) | 0 (0.0) | n.d. |  | 1 (0.8) | 0 (0.0) | 0 (0.0) | 0 (0.0) | 0 (0.0) | n.d. |
| *Sarcocystis* sp*.* | 1 (0.1) | 0 (0.0) | n.d. |  | 1 (0.1) | 0 (0.0) | n.d. |  | 0 (0.0) | 1 (0.1) | n.d. |  | 0 (0.0) | 0 (0.0) | 0 (0.0) | 1 (0.2) | 0 (0.0) | n.d. |
| *Cyclospora cayetanensis* | 1 (0.1) | 0 (0.0) | n.d. |  | 1 (0.1) | 0 (0.0) | n.d. |  | 1 (0.1) | 0 (0.0) | n.d. |  | 0 (0.0) | 0 (0.0) | 1 (0.6) | 0 (0.0) | 0 (0.0) | n.d. |
| *Hymenolepis nana* | 0 (0.0) | 1 (0.1) | n.d. |  | 1 (0.1) | 0 (0.0) | n.d. |  | 1 (0.1) | 0 (0.0) | n.d. |  | 0 (0.0) | 1 (0.9) | 0 (0.0) | 0 (0.0) | 0 (0.0) | n.d. |
| *Trichuris trichiura* | 0 (0.0) | 1 (0.1) | n.d. |  | 1 (0.1) | 0 (0.0) | n.d. |  | 1 (0.1) | 0 (0.0) | n.d. |  | 0 (0.0) | 1 (0.9) | 0 (0.0) | 0 (0.0) | 0 (0.0) | n.d. |

Data are presented as number of patients (percentage). Total number of patients is 1570 for sex and season, 1569 for age group (one birthdate lacking), and 1565 for geographical origin (five ZIP code lacking, see Fig 2 for the definition of North and South areas). p-values were calculated only for the most commonly represented parasites. To help reading, p-values <0.05 are indicated in bold. n.d.: not determined. **Enterocytozoon* *bieneusi* + *Encephalitozoon* sp..

## Discussion

Data about the prevalence of intestinal, protozoa, helminths and microsporidiosis in human populations from France and Luxembourg are scarce (21). The aim of this study was to provide an epidemiological update of intestinal parasitosis presence in individuals who had been prescribed a stool analysis for the presence of IPs in these two countries. One limitation of this study was the lack of clinical and epidemiological data, which were restricted to the age, sex and residence of the patients. Information such as eosinophilia, abdominal complaints, travel, or immunity status for example were not available owing to the outsourcing process of stool analysis. However, this enabled us to harvest at a large geographic scale numerous samples from all outpatients without distinction and for whom clinicians had requested a search for parasites. Stools were analysed by routine microscopy performed by experienced operators and several molecular assays to have as full an overview as possible. For example, *Sarcocystis* sp. is not targeted by any commercial PCR panel available to date, a drawback that reinforces the need to preserve microscopy skills or to push the manufacturers to develop new PCR for these targets (22).

Altogether, a parasite prevalence of 33.2% was found in the patients of this study. Of note, prevalence of pathogenic intestinal parasites was about 7%. Even though the presence of *Blastocystis* sp. and *D. fragilis* was reported in individuals with intestinal inflammation or irritable bowel syndrome, recent studies associated them to healthier microbiomes, suggesting a beneficial effect (23–28). However, it is important to emphasize that their high prevalence reflects faecal exposure.

The most prevalent parasite was *Blastocystis* sp., for which 20.5% of patients were positive. This is in accordance with figures from the most recent studies in France reporting 18.1% of *Blastocystis* sp. prevalence in a multicentre study involving 788 patients, and 10.5% in 488 patients and 14.5% in 186 patients in single-centre studies (2,5,11). Another previous study showed a seasonal pattern with more *Blastocystis* sp. in summer, a trend not confirmed herein (5). In addition, we observed no sex or geographical differences, and only children under five years old were less frequently positive for *Blastocystis* sp. compared with the other age groups (8.3% *vs* more than 16.6%, respectively). Different subtypes of *Blastocystis* sp., with some specific or zoonotic distribution among hosts, can be identified by sequencing of a 600-bp region of the gene encoding the 18S rRNA of the SSU rRNA (11). The most frequently found subtypes in our study were ST3, ST4, ST2 and ST1, all of which are frequently reported in humans, and in the same proportion in France as elsewhere in Europe (2,5,29,30). As previously reported, both ST6 and ST7, considered to be avian STs, were found more rarely (2,5). Interestingly, three patients were diagnosed with ST8, which is frequently reported in monkeys but rarely in humans. However, it was recently observed in abundance in wastewater samples in Denmark as a potential source of infection (29,30).

*Dientamoeba fragilis* was the second most commonly detected parasite, with a prevalence of 13.1% that varies from about 1% in Spain and Italy to more than 60% in northern Europe depending on the method used and the population studied (25). In France, data are scarce but three recent molecular prospective studies reported a prevalence of 2.3% in 488 patients, 11.9% from 588 samples (for 350 patients) and 6.5% in 673 patients (2,9,31). We observed a significantly higher prevalence in children aged 5-14 years (49.6%). The significantly higher prevalence in children under the age of 14 observed in this study is in line with the results of the Tchamwa Bamini *et al.* study (31). The reasons are uncertain but some authors have suggested that even in locations with high levels of sanitation, young children have lower hygiene practices than adults and are then predisposed to infections with enteric protozoa (26). We found a seasonality pattern of *D. fragilis* carriage with a higher prevalence in winter (15.0%) than in summer (11.0%). Greater promiscuity and increased human-to-human transmission in colder months could be the cause but this mode of transmission has yet to be clearly demonstrated for *D. fragilis* (32). Among the *D. fragilis* genotypes we found, as expected, almost exclusively genotype 1 (97.9%). Interestingly, three patients from different geographical areas were detected with genotype 2, which is rarely reported (25).

Prevalences were lower for pathogenic parasites such as *G. intestinalis* and *Cryptosporidium* sp. with an equal prevalence of 1.9% (n=30 patients), narrowly followed by 1.9% for *E. vermicularis* (n=29 patients). Frequent isolation of *G. intestinalis* is consistent with previous reports and, to a lesser extent, that of *E. vermicularis* (2,3,8,9). For *G. intestinalis*, a significantly higher prevalence was found in the South than in the North of the country, but without a clear explanation. It is known that giardiasis is associated with poor sanitary conditions and has a higher prevalence in developing countries, which does not seem to be the case in our study (33). As expected, we also found a trend (albeit non-significant) towards predominance of giardiasis in children (4.3% of 5-14 years old). Genotyping studies identified various assemblages, which differed from one another in host range. In our study, we found the two zoonotic assemblages A and B, mostly found in humans but also identified in various mammals. In most reports, especially in Europe, more patients are infected with assemblage B than assemblage A (34).

Interestingly, *Cryptosporidium* sp. was identified in our population sample as frequently as *G. intestinalis* and *E. vermicularis*, which confirms the status of *Cryptosporidium* sp. as a common pathogenic parasite in the general population. As mentioned above, our study also evidenced the seasonality of cryptosporidiosis, which had a 3-fold higher prevalence in summer than in winter (3.0% *vs* 1.0%), probably as the result of an increased consumption of raw vegetables and exposure to recreational waters, as previously reported (6). In the *Cryptosporidium* species, *C. parvum* is known to be zoonotic whereas *C. hominis* seems to be more specific, but not exclusively, to humans. Only these two species were detected in our study, which is consistent with the worldwide epidemiology of the parasite. Indeed, although a few other species are pathogenic for humans, *C. parvum* and *C. hominis* are involved in the large majority of human cases. This is also the case in France, as reported by Costa et al., who showed that 96% of human cases between 2017 and 2019 were caused by *C. parvum* (72%) and *C. hominis* (24%) (6). The genotyping results of *Cryptosporidium* from our sample populations are also consistent with the known European epidemiology of *Cryptosporidium*. In the subtype family of *C. parvum*, the IIa detected in our samples largely predominates in Europe, and is particularly associated with young cattle (35). Furthermore, the major subtype found in our study (n = 10) was IIaA15G2R1, which is the most widely distributed zoonotic subtype of *C. parvum* in France and in Europe (6,35). The subtype family of *C. hominis* that predominates in Europe is the Ib family, particularly represented by *C. hominis* IbA10G2. This subtype was not detected in our study, but the two subtypes we did detect, although less common, have already been described in calves and humans in France and in Europe (6,36,37). Interestingly, in a study dealing with *Cryptosporidium* diversity in Sweden, the IbA9G3 subtype was particularly associated with patients infected in Africa (36).

*Enterobius vermicularis* was identified with a relatively high prevalence in the present study even though stool specimens are not the samples of choice for this parasite, and a Graham Scotch tape test on the perianal skin should be preferred. Five cases were diagnosed by microscopy, the others by PCR only (Figure 4). As previously reported, there is a prevalence peak in the 5- to 14-year-old age class, which in our study was up to 7.0% (38). In the 29 cases detected, *E. vermicularis* was associated with *D. fragilis* in 18 cases. This high co-occurrence has already been reported leading to speculation about the carriage role of *E. vermicularis* ova in the transmission of *D. fragilis* (25).

As for *Cryptosporidium sp.*, microsporidia should be checked for in outpatients because it is not restricted to a specific population like HIV-positive patients or solid organ transplant recipients (39). A prevalence of 0.4% was found in our study making it a more common pathogen than *Taenia* sp. or *S. stercoralis*, for example. The genotypes we identified belong to the zoonotic Group 1 of *E. bieneusi*, which is consistent with the environmental reservoir and the faecal-oral route of transmission (40).

Altogether, amoeba prevalence reached 3.0%, with non-pathogenic species exclusively, most of which were *E. coli*, *E. nana* and *E. dispar* with some co-occurrence. Interestingly, no *E. histolytica* was identified in the study, which is consistent with data from previous French studies of hospitalized patients, which reported between 0 and 1.0% positive samples (2,8,9). Most of the cases diagnosed in France originate abroad. Unfortunately, we do not have access to the patients’ travel history in our study.

It is noteworthy that a relatively high number of patients had parasite co-occurrence. Almost 10% of patients carried two or more parasites, with certain preferential associations such as *Blastocystis* sp./*D. fragilis*, *D. fragilis*/*E. vermicularis* and *Blastocystis* sp./*G. intestinalis*, or harboured non-pathogenic intestinal amoebae *(E. coli*, *E. nana*, *E. dispar*, *Iodamoeba butschlii*, *Entamoeba hartmanni)* indicating a direct or indirect faecal-oral route of contamination. The age group 5-14 years was significantly more co-infected than others (S1 Table). This is explained by the main coinfection, *Blastocystis* sp./*D. fragilis*, which show the same difference in age class repartition for each of them. These findings show that multiple infections are not rare and could be even more common when bacteria and viruses are taken into consideration, as described in recent studies (41).

Finally, a comparison between PCR and microscopy, although this study was not designed for it, shows some preliminary data that will be interesting to develop in future studies. The use of PCR diagnostics increases the proportion of positive patients and strengthens the statistical significance for some variables (e.g., *Giardia* and region or *Cryptosporidium* and season). The prevalence increases significantly with the multiplication of samples by microscopy as well as by PCR, but not for all parasites. In positive patients with multiple stool samples, not all parasites were always detected in each sample, even with the PCR method. Thus the increased sensitivity of molecular assays does not make it possible to avoid repeated sampling for parasitological testing.

In conclusion, a high prevalence of intestinal parasites was found in France and Luxembourg with almost one third of patients being positive for at least one parasite. The most frequently detected parasites were *Blastocystis* sp. and *D. fragilis.* The high prevalence of pathogenic intestinal parasites (about 7%) highlights the importance of investigating gastrointestinal complaints with parasitic screening. In addition, co-occurrence of two or more parasites in a significant proportion of the population should encourage the use of the most exhaustive testing methods. Detailed data up to the genotyping level for multiple parasites provided in this study should encourage further research. The detection of parasites, of proven pathogenicity or not, remains a marker of faecal-oral route exposure and results should be interpreted accordingly. Clinicians should be familiar this epidemiology and be aware that industrialized countries are also affected by intestinal parasitic infection.

**Acknowledgements**

We thank all skilled microscopic operators that prepared and read all samples especially Gaelle Castan, Gwenaelle Guillet, Nathalie Joaquim, Xavier Sansico and Régine Serieys; and all students that helped to prepare samples (Joao Almeida, Chloé Belot, Issam Ben Rhouma, Farah Boughanmi, Apolline Riffard and Antoine Tranquy).

## References

1.	Fletcher SM, Stark D, Harkness J, Ellis J. Enteric Protozoa in the Developed World: a Public Health Perspective. Clin Microbiol Rev. 2012;25(3):420‑49.

2.	Menu E, Mary C, Toga I, Raoult D, Ranque S, Bittar F. A hospital qPCR-based survey of 10 gastrointestinal parasites in routine diagnostic screening, Marseille, France. Epidemiol Infect. 2019;147:e100.

3.	Masucci L, Graffeo R, Bani S, Bugli F, Boccia S, Nicolotti N, *et al*. Intestinal parasites isolated in a large teaching hospital, Italy, 1 May 2006 to 31 December 2008. Eurosurveillance. 2011;16(24):19891.

4.	Calderaro A, Montecchini S, Rossi S, Gorrini C, De Conto F, Medici MC, *et al*. Intestinal parasitoses in a tertiary-care hospital located in a non-endemic setting during 2006–2010. BMC Infect Dis. 2014;14(1):264.

5.	El Safadi D, Cian A, Nourrisson C, Pereira B, Morelle C, Bastien P, *et al*. Prevalence, risk factors for infection and subtype distribution of the intestinal parasite *Blastocystis* sp. from a large-scale multi-center study in France. BMC Infect Dis. 2016;16(1):451.

6.	Costa D, Razakandrainibe R, Valot S, Vannier M, Sautour M, Basmaciyan L, *et al*. Epidemiology of Cryptosporidiosis in France from 2017 to 2019. Microorganisms. 2020;8(9):1358.

7.	Kantzanou M, Karalexi MA, Vrioni G, Tsakris A. Prevalence of Intestinal Parasitic Infections among Children in Europe over the Last Five Years. Trop Med Infect Dis. 2021;6(3):160.

8.	Hartuis S, Lavergne RA, Nourrisson C, Verweij J, Desoubeaux G, Lussac-Sorton F, *et al*. The Novodiag Stool parasites assay, an innovative high-plex technique for fast detection of protozoa, helminths and microsporidia in stool samples: a retrospective and prospective study. Parasite. 2022;29:27.

9.	Autier B, Gangneux JP, Robert-Gangneux F. Evaluation of the Allplex Gastrointestinal Panel—Parasite Assay for Protozoa Detection in Stool Samples: A Retrospective and Prospective Study. Microorganisms. 2020;8(4):569.

10.	Hannet I, Engsbro AL, Pareja J, Schneider UV, Lisby JG, Pružinec-Popović B, *et al*. Multicenter evaluation of the new QIAstat Gastrointestinal Panel for the rapid syndromic testing of acute gastroenteritis. Eur J Clin Microbiol Infect Dis. 2019;38(11):2103‑12.

11.	Poirier P, Wawrzyniak I, Albert A, El Alaoui H, Delbac F, Livrelli V. Development and Evaluation of a Real-Time PCR Assay for Detection and Quantification of *Blastocystis* Parasites in Human Stool Samples: Prospective Study of Patients with Hematological Malignancies. J Clin Microbiol. 2011;49(3):975‑83.

12.	Peek R, Reedeker FR, van Gool T. Direct Amplification and Genotyping of *Dientamoeba* *fragilis* from Human Stool Specimens. J Clin Microbiol. 2004;42(2):631‑5.

13.	Brunet J, Lemoine JP, Pesson B, Valot S, Sautour M, Dalle F, *et al*. Ruling out nosocomial transmission of *Cryptosporidium* in a renal transplantation unit: case report. BMC Infect Dis. 2016;16:363.

14.	Bailly E, Valot S, Vincent A, Duffourd Y, Grangier N, Chevarin M, *et al*. Evaluation of Next-Generation Sequencing Applied to *Cryptosporidium* *parvum* and *Cryptosporidium* *hominis* Epidemiological Study. Pathogens. 2022;11(8):938.

15.	Hopkins RM, Meloni BP, Groth DM, Wetherall JD, Reynoldson JA, Thompson RC. Ribosomal RNA sequencing reveals differences between the genotypes of *Giardia* isolates recovered from humans and dogs living in the same locality. J Parasitol. 1997;83(1):44‑51.

16.	Lee SU, Chun HC, Huh S. Molecular phylogeny of parasitic Platyhelminthes based on sequences of partial 28S rDNA D1 and mitochondrial cytochrome c oxidase subunit I. Korean J Parasitol. 2007;45(3):181‑9.

17.	Sulaiman IM, Fayer R, Lal AA, Trout JM, Schaefer FW, Xiao L. Molecular Characterization of Microsporidia Indicates that Wild Mammals Harbor Host-Adapted *Enterocytozoon* spp. as well as Human-Pathogenic *Enterocytozoon* *bieneusi*. Appl Environ Microbiol. 2003;69(8):4495‑501.

18.	Katzwinkel‐Wladarsch S, Lieb M, Heise W, Löscher T, Rinder H. Direct amplification and species determination of microsporidian DNA from stool specimens. Trop Med Int Health. 1996;1(3):373‑8.

19.	Rubiola S, Civera T, Ferroglio E, Zanet S, Zaccaria T, Brossa S, *et al*. Molecular differentiation of cattle *Sarcocystis* spp. by multiplex PCR targeting 18S and COI genes following identification of *Sarcocystis* *hominis* in human stool samples. Food Waterborne Parasitol. 2020;18:e00074.

20.	Feise RJ. Do multiple outcome measures require p-value adjustment? BMC Med Res Methodol. 2002;2(1):8.

21.	Polage CR, Stoddard GJ, Rolfs RT, Petti CA. Physician Use of Parasite Tests in the United States from 1997 to 2006 and in a Utah *Cryptosporidium* Outbreak in 2007. J Clin Microbiol. 2011;49(2):591‑6.

22.	Bradbury RS, Sapp SGH, Potters I, Mathison BA, Frean J, Mewara A, *et al*. Where Have All the Diagnostic Morphological Parasitologists Gone? J Clin Microbiol. 2022;60(11):e0098622.

23.	Piperni E, Nguyen LH, Manghi P, Kim H, Pasolli E, Andreu-Sánchez S, *et al*. Intestinal *Blastocystis* is linked to healthier diets and more favorable cardiometabolic outcomes in 56,989 individuals from 32 countries. Cell. 2024;187(17):4554-4570.e18.

24.	Muhsin-Sharafaldine MR, Abdel Rahman L, Suwanarusk R, Grant J, Parslow G, French N, et al. *Dientamoeba fragilis* associated with microbiome diversity changes in acute gastroenteritis patients. Parasitol Int. 2023;97:102788.

25.	Stark D, Barratt J, Chan D, Ellis JT. *Dientamoeba* *fragilis*, the Neglected Trichomonad of the Human Bowel. Clin Microbiol Rev. 2016;29(3):553‑80.

26.	Barratt JLN, Harkness J, Marriott D, Ellis JT, Stark D. A review of *Dientamoeba* *fragilis* carriage in humans: Several reasons why this organism should be considered in the diagnosis of gastrointestinal illness. Gut Microbes. 2011;2(1):3‑12.

27.	Wawrzyniak I, Poirier P, Viscogliosi E, Dionigia M, Texier C, Delbac F, *et al*. *Blastocystis*, an unrecognized parasite: an overview of pathogenesis and diagnosis. Ther Adv Infect Dis. 2013;1(5):167‑78.

28.	Nourrisson C, Scanzi J, Pereira B, NkoudMongo C, Wawrzyniak I, Cian A, *et al*. *Blastocystis* is associated with decrease of fecal microbiota protective bacteria: comparative analysis between patients with irritable bowel syndrome and control subjects. PloS One. 2014;9(11):e111868.

29.	Meloni D, Sanciu G, Poirier P, El Alaoui H, Chabé M, Delhaes L, *et al*. Molecular subtyping of *Blastocystis* sp. isolates from symptomatic patients in Italy. Parasitol Res. 2011;109(3):613‑9.

30.	Stensvold CR, Lebbad M, Hansen A, Beser J, Belkessa S, O’Brien Andersen L, *et al*. Differentiation of *Blastocystis* and parasitic archamoebids encountered in untreated wastewater samples by amplicon-based next-generation sequencing. Parasite Epidemiol Control. 2020;9:e00131.

31.	Tchamwa Bamini G, Charpentier E, Guemas E, Chauvin P, Fillaux J, Valentin A, *et al*. No evidence of pathogenicity of *Dientamoeba* *fragilis* following detection in stools: A case-control study. Parasite. 2024;31:40.

32.	Stark D, Roberts T, Marriott D, Harkness J, Ellis JT. Detection and Transmission of *Dientamoeba fragilis* from Environmental and Household Samples. Am J Trop Med Hyg. 2012;86(2):233‑6.

33.	Ryan UM, Feng Y, Fayer R, Xiao L. Taxonomy and molecular epidemiology of *Cryptosporidium* and *Giardia* – a 50 year perspective (1971–2021). Int J Parasitol. 2021;51(13):1099‑119.

34.	Xiao L, Feng Y. Molecular epidemiologic tools for waterborne pathogens *Cryptosporidium* spp. and *Giardia* *duodenalis*. Food Waterborne Parasitol. 2017;8‑9:14‑32.

35.	Cacciò SM, Chalmers RM. Human cryptosporidiosis in Europe. Clin Microbiol Infect. 2016;22(6):471‑80.

36.	Lebbad M, Winiecka-Krusnell J, Stensvold CR, Beser J. High Diversity of *Cryptosporidium* Species and Subtypes Identified in Cryptosporidiosis Acquired in Sweden and Abroad. Pathog Basel Switz. 2021;10(5):523.

37.	Razakandrainibe R, Diawara EHI, Costa D, Goff LL, Lemeteil D, Ballet JJ, *et al*. Common occurrence of *Cryptosporidium* *hominis* in asymptomatic and symptomatic calves in France. PLoS Negl Trop Dis. 2018;12(3):e0006355.

38.	Leung AKC, Lam JM, Barankin B, Wong AHC, Leong KF, Hon KL. Pinworm (*Enterobius* *vermicularis*) Infestation: An Updated Review. Curr Pediatr Rev. 2024;

39.	Didier ES, Weiss LM. Microsporidiosis: Not just in AIDS patients. Curr Opin Infect Dis. 2011;24(5):490‑5.

40.	Li W, Feng Y, Santin M. Host Specificity of *Enterocytozoon bieneusi* and Public Health Implications. Trends Parasitol. 2019;35(6):436‑51.

41.	Mallier C, Creuzet E, Lambert C, Delmas J, Mirand A, Rochette E, *et al*. Summer diarrhea in children: a monocentric French epidemiological observational study. Sci Rep. 2023;13:15078.

**List of legends**

**S1 Table. Parasites co-occurrence by microscopy and/or molecular biology according to sex, geographical origin, season of sampling and age.**

**S2 Table. Prevalence by parasite according to the diagnostic method used.**

**S3 Table. Parasite and microsporidiosis prevalence by microscopy and molecular biology according to the number of stool samples per patient.**

**S4 Table. Number of stool samples required to detect all co-occurrences in the subset of patients with three stool samples according to the diagnostic method used.**

**S5 Table. Multivariable analysis of Blastocystis sp. and Dientamoeba fragilis carriage.**

**S6 Table. Parasites and microsporidia prevalence by microscopy and molecular biology according to sex, geographical origin, season of sampling and age.**

**S1 Dataset. Deidentified raw data used in the analysis.**
