# Supplementary material for: Current status of intestinal parasitosis and microsporidiosis in industrialized countries: Results from a prospective study in France and Luxembourg
Source: PLoS Negl Trop Dis. 2024 Dec 23;18(12):e0012752. doi: 10.1371/journal.pntd.0012752 (PMC11706478; doi:10.1371/journal.pntd.0012752)
Supplement: Attachment — Submitted filename: Response to Reviewers.docx [file pntd.0012752.s010.docx]

<https://www.google.fr/url?sa=i&url=https://twitter.com/chuclermontfd?lang%3Deu&psig=AOvVaw2bG-aOKDB22am21JcCrsHh&ust=1610965014361000&source=images&cd=vfe&ved=0CAIQjRxqFwoTCIjTu73eou4CFQAAAAAdAAAAABAE>22 November 2024

Response to Editor

**SERVICE DE**

**PARASITOLOGIE-MYCOLOGIE**

***C****entre* ***N****ational de* ***R****éférence*

*des* ***C****ryptosporidioses,* ***M****icrosporidies*

*et* ***A****utres* ***P****rotozooses digestives (****CMAP****)*

**Pr Philippe POIRIER - Chef de Service**

Professeur des Universités – Praticien Hospitalier

[ppoirier@chu-clermontferrand.fr](mailto:ppoirier@chu-clermontferrand.fr)

**Mme Magali BOURG - Cadre de Santé**

mbourg@chu-clermontferrand.fr

**Mme Carine BONALDI - Secrétaire**

[cbonaldi@chu-clermontferrand.fr](mailto:cbonaldi@chu-clermontferrand.fr)

**Dr Céline NOURRISSON**

Maître de Conférences des Universités – Praticien Hospitalier

[c_nourrisson@chu-clermontferrand.fr](mailto:c_nourrisson@chu-clermontferrand.fr)

**Pr Valérie LIVRELLI**

Professeur des Universités

Praticien Attaché

[vlivrelli@chu-clermontferrand.fr](mailto:vlivrelli@chu-clermontferrand.fr)

**Dr Maxime MONIOT**

Praticien Hospitalier

[mmoniot@chu-clermontferrand.fr](mailto:mmoniot@chu-clermontferrand.fr)

**Mme Patricia COMBES**

Ingénieure CNR CMAP

pcombes@chu-clermontferrand.fr

Service de Parasitologie-Mycologie
Centre de biologie (5ème étage)
CHU Gabriel Montpied
58 rue Montalembert
63003 Clermont-Ferrand
Cedex 1

Tel: 33 (0)4 73 75 01 95
Fax: 33 (0)4 73 75 01 96

Dear Editor,

Please find attached the revised manuscript. As expected, we have addressed below each of the issues in a point-by-point reply (in bold). The modifications in the new version of the manuscript are highlighted in yellow.

We hope that changes that have been made in our amended manuscript will answer to the editor queries and that you will find our article suitable for publication in your journal. All authors agree with this new version of our manuscript.

Yours faithfully,

Pr Philippe Poirier

**Additional Editor Comments (if provided):**

The authors have modified and revised the manuscript according to the reviewers suggestions. Nonetheless, I have revised the re-submitted manuscript and have some minor comments that need to be addressed before we can accept the study for publication.

The population of the study is unclear, throughout the manuscript the authors refer to the study population as primary care patients, primary care, community based care patients, outpatients, community care, etc. This needs to be uniform throughout and clearly explained in the method section, it's still not clear to me where the stool samples came from.

**We agree that this could be confusing. So, we have added the following precision in the method section, lines 113-119: “The samples came from primary health care laboratories that did not perform intestinal parasite analysis and outsourced it to our laboratory at the Clermont-Ferrand University Hospital Center. […] The study population was therefore primary care patients with a prescription for IPs testing. Clinical data were not available, but the following patient information was provided with the samples: sex, age and ZIP code of residence.”**

**Line 48: we have replaced “community-based care” by “primary health care”.**

**Line 52: we have replaced “community-based care” by “primary health care”.**

**Line 54: we have added the following precision “primary health care”.**

**Line 74: we have replaced “community-based care” by “primary health care”.**

**Line 76: we have replaced “community-based care” by “primary health care”.**

**Line 89: we have replaced “community-based care” by “primary health care”.**

**Line 96: we have replaced “outpatients” by “primary health care”.**

**Line 105: we have replaced “community care” by “primary health care”.**

**Line 312: we have replaced “all outpatients” by “primary health care patients”.**

**Line 364: we have replaced “outpatients” by “primary health care patients”.**

Also, in some sections the authors mention co-occurrence of parasites and in other co-infection, I suggest using co-infection.

**We have replaced the term "co-occurrence" with the term "coinfection" lines 62, 80, 122, 268, 346, 369, 392, 526, 534.**

Finally, the discussion is too long and drawn out, please try to shorten in by providing more concise comparison with other studies.

**We have shortened the discussion (by removing 597 words) and kept only 5 paragraphs:**

- **strengths and weaknesses of the study**
- **non-pathogenic parasites**
- **pathogenic parasites**
- **coinfections**
- **comparison PCR / microscopy**

I have also added specific comments and corrections to the attached file.

**Specific comments and corrections have been taken into account.**

**We have added the following sentence in the abstract line 68: “The detection of parasites, pathogenic or not, remains a marker of the faecal-oral route of transmission and results should be interpreted accordingly.” This notion was already present in author summary line 83, discussion lines 319 and conclusion line 395.**

**In the introduction, we have clarified the objective of the study. We have also moved** **inclusion and exclusion parameters in the method section.**

**In the methods section, the paragraph on clinical samples has been clarified.**

**We have moved “five ZIP code lacking” from the legend to the relevant paragraph line 199.**

**Line 313:** **we have added the following precision “analyzed in a single center”.**

**Reviewers' comments:**

None
